# Androgen receptor expression identifies patient with favorable outcome in operable triple negative breast cancer

**DOI:** 10.18632/oncotarget.16913

**Published:** 2017-04-07

**Authors:** Xiao-Qing Hu, Wei-Li Chen, Hai-Guang Ma, Ke Jiang

**Affiliations:** ^1^ Department of Surgical Oncology, Wenzhou Central Hospital, Zhejiang, China; ^2^ Department of Breast Surgery, Yue Yang Hospital of Traditional Chinese & Western Medicine, Shanghai University of Traditional Chinese Medicine, Shanghai, China

**Keywords:** androgen receptor, triple negative breast cancer, prognostic biomarker, disease-free survival, overall survival

## Abstract

In this study we sought to investigate the prevalence and prognostic value of androgen receptor (AR) status in operable triple-negative breast cancer (TNBC) patients. We collected the clinical data of 360 patients with TNBC, and found a positivity AR expression of 31.4% with a cut-off value of 10%. Tumors expressing the negative CK5/6 (P=0.013) and low Ki-67 (P=0.007) are more likely to have positive AR. In multivariate survival analysis, AR expression is correlated with increased DFS (HR=0.467, 95%CI 0.271-0.805; P=0.006) and OS (HR=0.488, 95%CI 0.267-0.894, P=0.020) independently. In addition, patients with AR+ tumors are more likely to have favorable outcome in patients with young, pre-menopausal, large tumor size, more node involvement (4+), high stage, high grade, vascular invasion+, P53+, CK5/6-, and higher Ki-67. Our study has indicated that the absence of AR might help to identify patients with relatively higher risk of disease relapse and death, and further clinical studies of anti-androgen agents are warranted to enrich the therapeutic strategy options for AR+ TNBCs.

## INTRODUCTION

Breast cancer is a heterogeneous disease with several distinct subtypes that are based on differential patterns of gene expression. In recent years, breast cancers have been classified into four subtypes (luminal A, luminal B, HER-2 positive, and triple-negative) according to the status of hormonal receptors, Ki-67 and human epidermal growth factor receptor-2 (HER-2) expression [1]. Triple-negative breast cancer (TNBC), which lacks expression of estrogen receptor (ER), progesterone receptor (PR) and HER-2, is believed to have a relatively aggressive tumor biology [2]. Patients with TNBC have significantly worse prognosis compared to other breast cancer subtypes due to the lack of well-defined targeted molecular therapy [3].

Previous reports showed that TNBC could be classified into 7 subtypes by gene expression microarray [4, 5], indicated that TNBC is a heterogeneous disease comprising subtypes with different biological behaviors and treatment responses. It is recently reported that the seven-subtype system have been further modified [6]. However, compared to immunohistochemistry (IHC), gene-expression profiling is still premature in clinical practice. Thus, surrogate IHC markers have served as a more practical means of assessing preclinical and clinical predictive effects on tumor characteristics and patient outcome. For instance, androgen receptor (AR) could be detected by IHC to identify TNBC subset referred to as luminal androgen receptor subtype (LAR) [4].

Depending on the thresholds of positivity used, AR is expressed in 10-53% of TNBC, and positivity of only AR is far more common in patients than positivity in only ER, PR or HER-2 [7, 8]. The prognostic value of AR in TNBC varies among literatures. A meta-analysis included 2826 TNBC patients suggested that absence of AR expression in TNBC served as a high-risk factor for both disease recurrence and death [9]. On the contrary, some other studies revealed that AR+ TNBC had worse survival [4, 10, 11]. In this study, we aimed to investigate the prevalence and prognostic value of AR status with a multicenter experience of Chinese women with TNBC.

## RESULTS

### Patients’ characteristics

A total of 360 patients with TNBC were enrolled in this study. The characteristics of the patients are shown in Table 1. Among 360 patients, the median age was 52 (ranges 21-89), and

**Table 1:** Characteristics of patients according to AR status (n=360)

Characteristics	Number of patients			P value
	All	AR- (%)	AR+ (%)	
**Age**				0.557
<40	41	30 (73.2%)	11 (26.8%)	
40-60	228	154 (67.5%)	74 (32.5%)	
>60	91	58 (63.7)	33 (36.3%)	
**Menopausal status**				**0.037**
Pre	150	110 (73.3%)	40 (26.7%)	
Post	210	132 (62.9%)	78 (37.1%)	
**Tumor size**				0.133
<2cm	184	117 (63.6%)	67 (36.4%)	
2-5cm	176	125 (71.0%)	51 (29.0%)	
**Node status**				0.561
0	227	148 (65.2%)	79 (34.8%)	
1-3	74	52 (70.3%)	22 (29.7%)	
4+	59	42 (71.2%)	17 (28.8%)	
**Stage**				0.166
I	125	76 (60.8%)	49 (39.2%)	
II	176	124 (70.5%)	52 (29.5%)	
III	59	42 (71.2%)	17 (28.8%)	
**Grade**				**0.007**
I-II	186	137 (73.7%)	49 (26.3%)	
III	174	105 (60.3%)	69 (39.7%)	
**Vascular invasion**				0.748
-	277	185 (66.8%)	92 (33.2%)	
+	83	57 (68.7%)	26 (31.3%)	
**P53**				**0.013**
-	143	107 (74.8%)	36 (25.2%)	
+	217	135 (62.2%)	82 (37.8%)	
**CK5/6**				**0.013**
-	214	133 (62.1%)	81 (37.9%)	
+	146	109 (74.7%)	37 (25.3%)	
**Ki-67**				**0.007**
<15%	83	57 (68.7%)	26 (31.3%)	
15%-50%	129	74 (57.4%)	55 (42.6%)	
50%-100%	148	111 (75%)	37 (25.0%)	
**Operation**				0.932
BCS	25	17 (68.0%)	8 (32.0%)	
Mastectomy	335	225 (67.2%)	110 (32.8%)	
**Chemotherapy**				0.550
None	26	20 (76.9%)	6 (23.1%)	
CEF	146	97 (66.4%)	49 (33.6%)	
CEF-T	188	125 (66.5%)	63 (33.5%)	
**Radiotherapy**				0.089
Yes	113	83 (73.5%)	30 (26.5%)	
No	247	159 (64.4%)	88 (35.6%)	
**All**	360	247 (68.6%)	113 (31.4%)	

AR, androgen receptor; BCS, breast conserving surgery; CEF, cyclophosphamide+epirubicin+5-fluorouracil; CEF-T, cyclophosphamide+epirubicin+5-fluorouracil followed by taxol.

41.7% of them were pre-menopausal. 125 patients were considered stage I disease, whereas 176 were considered Stage II and 59 were considered as stage III disease. Most of patients were diagnosed as invasive ductal carcinoma (344), whereas only 16 patients were of other histological types (7 metaplastic carcinoma, 3 lobular carcinoma, 3 micropapillary carcinoma, 1 medullary carcinoma, 1 mucinous adenocarcinoma and 1 adenocystic carcinoma). Majority of patients received mastectomy (335) and the other 25 patients received BCS. Chemotherapy was performed in 92.8% of patients, and radiation therapy was performed in 31.4% of patients. Among 360 patients, 113 (31.4%) was AR+. Table 1 shows the patient characteristics according to AR category. A higher proportion of positive AR were most likely to be observed in patients with post-menopausal status (P=0.037), grade III (P=0.007), P53+ (P=0.013), CK5/6- (P=0.013), and lower Ki-67 value (P=0.007). However, the expression of AR was not correlated to patient age, tumor size, node status, stage, and vascular invasion or treatment strategies.

### Survival and prognostic factors

**Figure 1:**
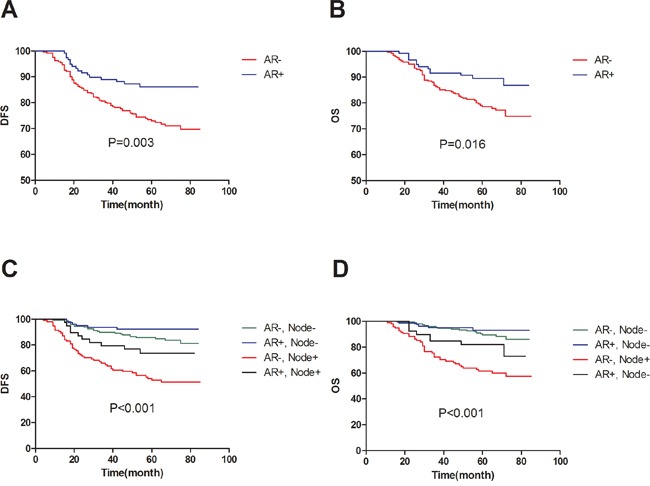
The distributions of the survival curves by androgen receptor in 360 TNBC patients **(A)** DFS according to patient category by AR; **(B)** OS according to patient category by AR; **(C)** DFS according to patient category by AR and node status; **(D)** OS according to patient category by AR and node status.

The median follow-up time was 64 months. For 360 patients, the 5-year DFS was 75% and the 5-year OS was 80%. Table 2 shows the result of univariate and multivariate survival analyses. Tumor size (P<0.001), node status (P<0.001), stage (P<0.001), grade (P=0.021), vascular invasion (P<0.001), AR (P=0.005), chemotherapy (P-=0.028), radiotherapy (P<0.001) were significant predictors of DFS and were entered into the multivariate Cox regression model with forward selection. Tumor size (P=0.002), node status (P<0.001), stage (P<0.001), vascular invasion (P<0.001), AR (P=0.019), radiotherapy (P<0.001) were also predictors of OS. In the multivariate Cox model, AR showed an independent prognostic value for both DFS (HR=0.467, 95%CI 0.271-0.805; P=0.006) and OS (HR=0.488, 95%CI 0.267-0.894, P=0.020). Node status was also an independent predictor of patient outcome (P<0.001 for both DFS and OS). Better survival was more frequently observed in patients with positive AR and fewer involved nodes. The distributions of the survival curves by AR are shown in Figure 1A and 1B (log-rank test, P=0.003 for DFS and P=0.016 for OS). Combined with node status (shown in Figure 1C and 1D), patients with AR+ and node- have favorable outcomes with an observed 5-year DFS of 92% and an observed 5-year OS of 93%. However, patients with AR- and node+ were at relatively higher risk of relapse and death (5-year DFS of 51% and 5-year OS of 60%).

**Table 2:** Univariate and multivariate survival analysis of TNBC patients (n=360)

Characteristics	N	Disease-free survival			Overall survival		
		Uv		Mv	Uv		Mv
		P	P	HR (95%CI)	P	P	HR (95%CI)
**Age**		0.982	-	-	0.972	-	-
<40	41						
40-60	228						
>60	91						
**Menopausal status**		0.671	-	-	0.878	-	-
Pre	150						
Post	210						
**Tumor size**		<0.001	0.018		0.002	NS	-
<2cm	184			Ref.			
2-5cm	176			1.731 (1.097-2.734)			
**Node status**		<0.001	<0.001		<0.001	<0.001	
0	227			Ref.			Ref.
1-3	74			1.858 (1.024-3.369)			1.992 (1019-3.893)
4+	59			6.362 (3.913-10.343)			7.685 (4.458-13.247)
**Stage**		<0.001	NS	-	<0.001	NS	-
I	125						
II	176						
III	59						
**Grade**		0.021	NS	-	0.111	-	-
I-II	186						
III	174						
**Vascular invasion**		<0.001	NS	-	<0.001	-	-
-	277						
+	83						
**P53**		0.158	-	-	0.713	-	-
-	143						
+	217						
**AR**		0.005	0.006		0.019	0.020	
-	242			Ref.			Ref.
+	118			0.467 (0.271-0.805)			0.488 (0.267-0.894)
**CK5/6**		0.552	-	-	0.505	-	-
-	214						
+	146						
**Ki-67**		0.599	-	-	0.735	-	-
<15%	83						
15%-50%	129						
50%-100%	148						
**Operation**		0.367	-	-	0.546	-	-
BCS	25						
Mastectomy	335						
**Chemotherapy**		0.028	NS	-	0.214	-	-
None	26						
CEF	146						
CEF-T	188						
**Radiotherapy**		<0.001	NS	-	<0.001	NS	-
Yes	113						
No	247						

Uv, univariate analysis; Mv, multivariate analysis; HR, hazard ratio; CI, confidence interval; NS, no significance; Ref, reference; AR, androgen receptor; BCS, breast conserving surgery; CEF, cyclophosphamide+epirubicin+5-fluorouracil; CEF-T, cyclophosphamide+epirubicin+5-fluorouracil followed by taxol.

We also investigated the prognostic value of AR in patient subgroups. Table 3A and 3B show the HR and 95%CI of AR, demonstrating the prognostic strength for DFS and OS. AR positivity was correlated to better survival compared to AR negativity in most subgroups. The prognostic value of AR were statistically significant in patients with age of 40-60, pre-menopausal status, large tumor size (2-5cm), more node involvement (4+), high stage (III), high grade (III), vascular invasion+, P53+, CK5/6-, and higher Ki-67 (50%-100%).

**Table 3A:** Hazard ratio of overall survival according to AR status in patient subgroups

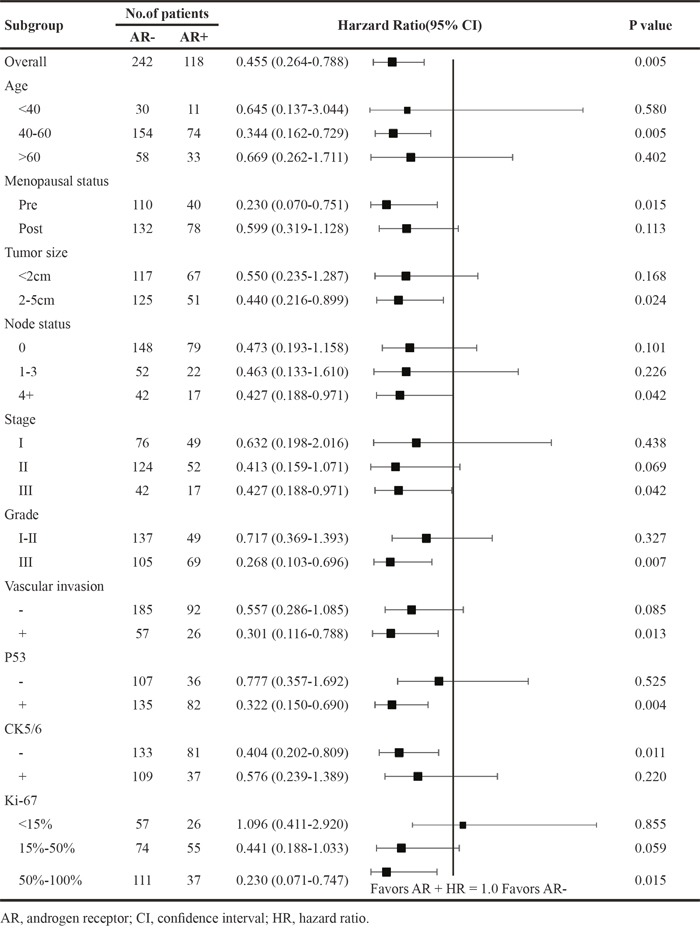

**Table 3B:** Hazard ratio of overall survival according to AR status in patient subgroups

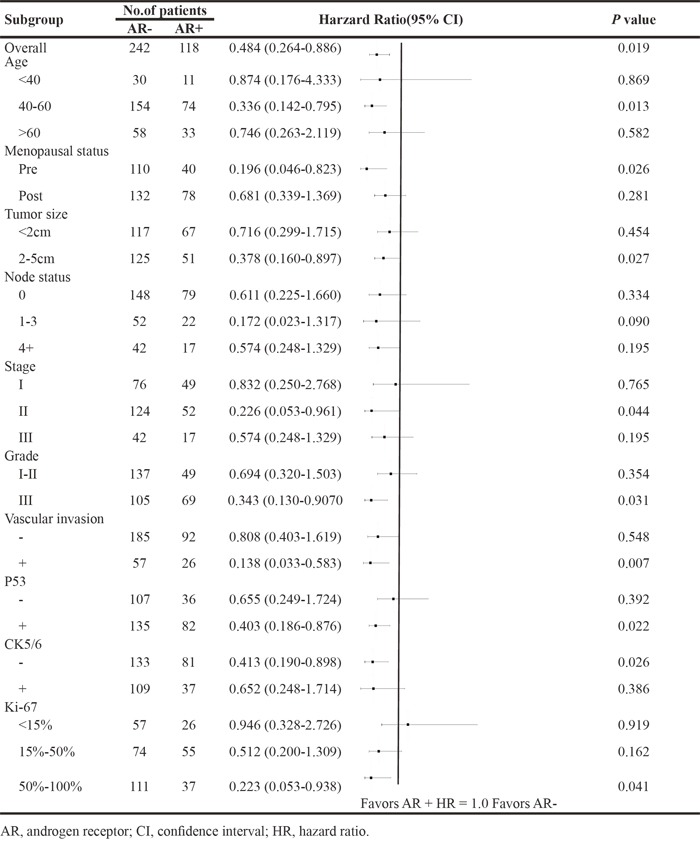

## DISCUSSION

Breast cancer has long been recognized as a heterogeneous disease. While the intrinsic subtypes of breast cancer based on gene array analysis has already been discussed, the IHC detection of receptor status is regarded as the most useful method in predicting prognosis and responsiveness to treatment. Breast cancer that lacks ER, PR and overexpression of HER-2, known as TNBC, is not amenable to the currently available targeted therapies and has a poor prognosis. Compared with other breast cancer subtypes, TNBC has a higher response rate to neoadjuvant chemotherapy; however, this advantage is not clearly translated into an improved overall survival [12]. This so-called TNBC paradox might be related to the heterogeneity of TNBC, and has attracted great attention of clinicians and researchers. Treating TNBC has always been challenging because of the heterogeneity and the absence of well-defined molecular targets. Previous reports showed that TNBC could be classified into 7 subtypes by gene expression microarray including two basal-like (BL1 and BL2), an immunomodulatory (IM), a mesenchymal (M), a mesenchymal stem-like (MSL), a luminal androgen receptor (LAR) and an unstable (UNS) subtype [4]. One of a well-known subtype, the LAR is heavily enriched in hormonally regulated pathways showed less response to chemotherapy and delayed recurrences compared with the other groups [5], suggesting that it has a clearly different clinical process.

One of the main characteristics of LAR is the expression of AR, a hormonal receptor that can be evaluated through IHC. AR is a commonly expressed biomarker in both normal breast and breast cancer tissues. In TNBC, the prevalence of AR positivity varies among literatures, depending on different thresholds of positivity. While some studies use a cut-off value of 1% staining [10, 13], many studies use a cut-off value of 5% [14] or 10% [15–17]. In this study, we found AR positivity in 31.4% of patients with a cut-off value of 10%. Remarkably, higher proportion of positive AR were most likely observed in patients with CK5/6-. Since CK5/6 is a well-known biomarker of basal-like subtype (BLBC), our study is consistent with previous studies that basal-like TNBCs have the least expression of AR [8]. Furthermore, we also found that AR positivity is associated with a lower Ki-67 index. It might be an indication of why non-basal-like TNBC responds less well than basal-like TNBC to chemotherapy, as discussed previously [16, 18]. Taken together, the expression of AR might identify certain TNBC molecular subtypes.

The prognostic value of AR still remains controversial. Some studies showed that TNBC patients with positive AR expression having poor overall survival [10, 14, 19], however, some other study drawn opposite conclusion [14]. A recent meta-analysis included 2826 TNBC patients showed an association of improved survival outcomes and AR expression in patients with TNBC [9]. In agreement with this study, we have demonstrated that AR showed an independent prognostic value for both DFS (HR=0.467) and OS (HR=0.488). Patients with AR+ and node- has favorable outcome with an observed 5-year DFS of 92% and an observed 5-year OS of 93%, which is almost 1.5-2 times higher than patients with AR- and node+. Furthermore, in subgroup analyses, AR positivity was significantly correlated to a better survival in patients with young, pre-menopausal, large tumor size, more node involvement (4+), high stage, high grade, vascular invasion+, P53+, CK5/6-, and higher Ki-67. Our findings have indicated that expression of AR reveals tumor of better biological behavior in high-risk TNBC. The contradictory result of prognostic value of AR might correlate to the potential antagonizing effect of AR against ER signaling. It is believed that without functional ER, AR may be the primary driver that facilitate cancer progression [20]. It is reported that with ER expression less than 10%, AR expression incurred better prognosis irrespective of ER co-expression [21]. Since cut-off point of this study is only 1%, the mutual influence of ER versus AR could be negligible which makes our study reliable. Furthermore, one other possible explanation is the different cutoff value of AR. In Wang's meta-analysis, the cutoff value for AR positivity was reported in 10 (76.9%) of 12 studies: one study (7.7%) used 0%, three studies (23.1%) used 1%, one study (7.7%) used 5%, and four studies (30.8%) used 10%. Remarkably, the low cut-offs (1% or 5%) or high cut-offs used in different studies did not affected the prognostic value of AR. In our study, we tried 1%, 5% and 10% as cutoff values. However, only about 10% of patients were consider as 1%-5% positivity of AR, and the different cut-offs did not affect the main conclusion of this study. In consistent with most studies, we choose 10% as the cutoff of AR in this manuscript. Another interference factor is the regimens of adjuvant chemotherapy for TNBCs. CEF and CEF-T were used in this study according to guideline at the treating time; however, the dose-dense AC followed by paclitaxel chemotherapy might be more suitable for TNBC according to present version of NCCN guideline. Since there were not survival difference in patients treated with CEF or CEF-T, we believed that the treatment regimen will not influence the prognostic value of AR.

The expression of AR is associated with a luminal subtype as determined by gene microarray, while the majority of AR negative TNBC exhibit a basal-like molecular subtype. In fact, gene-expression profiling has not fully replaced classical IHC yet, since it is not a routine practice. Thus, surrogate IHC markers have served as a more practical means of a preclinical and clinical predictive effect on patient outcome and differential response to specific agents. AR- TNBC, also known as quadruple negative breast cancer (QNBC), has revealed a different gene expression compared with AR+ TNBC [8, 22]. Our study has also provided clinical evidence highlighting the necessity of redefining TNBC as AR+ or QNBC. More importantly, AR has recently been developed as a potential therapeutic target. Recently, a phase II trial of bicalutamide, an androgen antagonist, has shown a clinical benefit rate of 19% in a select group of patients with ER/PgR-negative, AR-positive breast cancer [23]. Other studies and new agents of androgen blockade might also play important roles in developing new therapeutic strategy for TNBC (such as NCT00468715, NCT00516542, NCT01597193).

In conclusion, we have provided another evidence of the relationship between AR and patient survival in TNBC. The redefinition of QNBC that distinguished from TNBC by the absence of AR might help to identify patients with relatively higher risk of disease relapse and death. For AR+ TNBC, the developing of anti-androgen treatment might help to enrich therapeutic strategy for TNBC in clinical practice, however, it is still far from mature. Future studies refer to gene expression, signal pathways and biomarkers in different subtypes of TNBC are required for developing new therapeutic targets.

## PATIENTS AND METHODS

### Study population

From January 2009 to December 2011, 2143 patients with operable invasive breast cancer received curative surgery at Wenzhou Central Hospital and Yue Yang Hospital of Traditional Chinese&Western Medicine. We retrospectively collected data of clinical and pathological characteristics from 360 consecutive cases diagnosed with TNBC confirmed by immunohistochemistry (IHC) test. Since most of the patients with T3-T4 primary tumor underwent neoadjuvant chemotherapy in our centers, only T1-T2 disease were included in this study. Other exclusion criteria included bilateral breast cancer and male breast cancer.

### Treatment and follow up

Mastectomy or BCS was performed to all patients in this study. Axillary node involvement was evaluated by sentinel node biopsy or axillary lymph node dissection at the discretion of treating surgeon according to NCCN guideline. For most of the patients, adjuvant chemotherapy were administered within one month after operation with a total of 6–8 cycles. The adjuvant chemotherapy regimens include CEF (cyclophosphamide+epirubicin+5-fluorouracil), and CEF-T, (cyclophosphamide+epirubicin+5-fluorouracil followed by taxol) with standard dosage according to NCCN guideline. Radiation therapy was offered according to the treating radiation oncologist. All patients were followed-up every 3 months for the first year and then every 6 months until death.

### Immunohistochemistry

Immunohistochemistry (IHC) was performed on formalin-fixed, paraffin-embedded tissue sections from tumor specimens using standard procedures to evaluate biomarkers, including ER, PR, HER-2, Ki-67, P53, CK5/6 and AR. The cut-off value for ER positivity and PR positivity was 1% of tumor cells with positive nuclear staining. HER-2 was evaluated as 0, 1+, 2+ or 3+ using circumferential membrane-bound staining [HercepTest (Dako Cytomation, Carpinteria, CA, US)]. Positivity for HER-2 (HER-2+) was considered as 3+ using IHC or as positive on fluorescence *in situ* hybridization (FISH), whereas cases with 0 to 1+ or 2+ using IHC but without FISH detection were regarded as negative for HER-2 (HER-2-). Only tumors that were ER-, PR- and HER-2- were considered as TNBC and were included in this study. The Ki-67 value was expressed as the percentage of positive cells (at least 1000) with nuclear staining in each case. CK5/6 was considered positive if 10% or more of the tumor cells showed positive membrane expression. The examination of P53 and AR expression was assessed on the basis of nuclear staining intensity, and more than 10% of tumor cell nuclei stained were considered positive.

### Statistics

The Chi-squared test was used to evaluate the relationship between the patient characteristics and AR status. Disease-free survival (DFS) was defined as the length of time from the date of operation to events (local relapse or distant metastasis disease, occurrence of a new primary tumor or death without evidence of cancer). OS was defined as the length of time from the date of operation to death. Patients without any event or death were censored at the time of last follow-up. Univariate and multivariate survival analyses were performed by using the Cox regression model. Hazard ratio (HR) and 95% confidence interval (CI) were presented. Survival curves were estimated using the Kaplan–Meier method, and the log-rank test was used to test for differences between groups. All P-values reported were two sided and were calculated at a significance level of 0.05. All statistical procedures were carried out using SPSS (version 13.0; SPSS Company, Chicago, IL).

## Abbreviations

AR: androgen receptor
TNBC: triple-negative breast cancer
HER-2: epidermal growth factor receptor-2
ER: expression of estrogen receptor
PR: progesterone receptor
IHC: immunohistochemistry
LAR: luminal androgen receptor
CEF: Cyclophosphamide+epirubicin+5-fluorouracil
CEF-T: Cyclophosphamide+epirubicin+5-fluorouracil followed by taxol
DFS: Disease-free survival
OS: overall survival
HR: Hazard ratio
CI: confidence interval
BL: basal-like
IM: immunomodulatory
M: mesenchymal
MSL: mesenchymal stem-like
UNS: unstable
QNBC: quadruple negative breast cancer
Wei-Li Chen: collected and analyzed the data
Hai-Guang Ma: statistics and edited table and figure
